# Decoding tRNA dynamics in neuroimmune disorders: mechanistic insights, diagnostic innovations, and therapeutic opportunities

**DOI:** 10.3389/fimmu.2025.1642370

**Published:** 2025-11-20

**Authors:** Enlin Liang, Wenya Wang, Li Zhang

**Affiliations:** 1 Department of Pediatrics, West China Second University Hospital, Sichuan University, Chengdu, China; 2 Key Laboratory of Birth Defects and Related Diseases of Women and Children, Sichuan University, Ministry of Education, Chengdu, China

**Keywords:** tRNA modifications, tsRNAs, neuroinflammation, autoimmune encephalopathy, biomarker discovery, precision immunology

## Abstract

Transfer RNA (tRNA) and its derivatives, once regarded solely as translational adaptors, are now recognized as pivotal regulators of neuroimmune homeostasis. Dysregulated tRNA biogenesis, stress-induced fragmentation, and chemical modifications are increasingly implicated in the pathogenesis of neuroinflammatory and neurodegenerative disorders, including multiple sclerosis, neuromyelitis optica spectrum disorder, Alzheimer’s disease, and Parkinson’s disease. This review synthesizes emerging evidence on tRNA-derived small RNAs (tsRNAs), tRNA-modifying enzymes, and mitochondrial tRNA variants as drivers of immune dysregulation, glial activation, and neuronal injury. We highlight innovative diagnostic biomarkers (e.g., plasma tsRNAs, aminoacyl-tRNA synthetase-interacting multifunctional protein 1) and therapeutic strategies targeting tRNA modification pathways (e.g., queuine analogs, tRNA ligase inhibitors). By bridging tRNA biology with neuroimmunology, this work underscores the translational potential of tRNA-centric approaches in managing complex neurological diseases.

## 1 Introduction: transfer RNA dynamics—from translational workhorse to neuroimmune regulator

Transfer RNA (tRNA) are generally recognized as essential mediators of genetic information flow from nucleic acids to proteins, acting as indispensable adaptors during protein synthesis (1). On average, a eukaryotic tRNA molecule contains approximately thirteen post-transcriptional modification sites (2). Appropriate tRNA modifications play a crucial role in maintaining translational stability (3). Beyond their canonical role in protein synthesis, tRNA and its derivatives are increasingly appreciated as dynamic regulators of cellular homeostasis, stress responses, immune functions, and neurodevelopment (4–7). Given the growing body of evidence linking tRNA and its derivatives to immune modulation and neural integrity, it is crucial to further investigate how they participate in neuroimmune disorders and neuroinflammation.

Notably, mutations in tRNA genes, abnormalities in tRNA modification enzymes, and altered expression of tRNA derivatives have all been implicated in the pathogenesis of a broad range of human diseases. These include not only classic neurodegenerative disorders such as Alzheimer’s disease (AD) and Parkinson’s disease (PD), but also neuroimmune disorders (8–10). Neuroimmune disorders are a group of nervous system disorders mediated by autoimmune mechanisms, representing a complex interplay between immune dysregulation and neural tissue damage (11). The major types of neuroimmune disorders include multiple sclerosis (MS), neuromyelitis optica spectrum disorder (NMOSD), autoimmune encephalitis, myasthenia gravis, Guillain-Barré syndrome. Among them, MS is the most common and is characterized primarily by central nervous system (CNS) demyelination (12). Recent studies have revealed disease-specific expression patterns and functional roles of tRNA and its derivatives in regulating immune cell activation, glial responses, and neuroinflammatory cascades in these disorders. As such, tRNA and its derivatives are increasingly being investigated as biomarkers detectable in biofluids such as blood and cerebrospinal fluid, and as potential targets for precision therapeutics aimed at restoring tRNA homeostasis (10, 13).

In this review, we systematically summarize the multifaceted roles of tRNA and its derivatives in the regulation of neuroimmune disorders and neuroinflammatory processes that underlie neurodegenerative diseases and other neurological diseases. We highlight recent mechanistic insights into how tRNA modification and metabolism intersect with immune modulation in diseases such as MS, NMOSD, AD, PD, amyotrophic lateral sclerosis (ALS), ischemic stroke, and viral encephalitis. We further discuss innovative diagnostic and therapeutic strategies targeting tRNA-related pathways, emphasizing their potential for treating neuroimmune disorders. Understanding the versatile functions of tRNA and its derivatives will not only expand our knowledge of neuroimmune pathology but may also pave the way for novel diagnostic and therapeutic approaches.

## 2 tRNA biogenesis, stress-induced fragmentation, and chemical modifications

### 2.1 Transcriptional regulation and maturation of tRNA

To better appreciate the involvement of tRNA metabolism in neuroimmune disorders, it is essential to first understand the basic biology of tRNA and its associated enzymatic systems. tRNA genes are transcribed by RNA polymerase III to produce a precursor molecule (pre-tRNA), which undergoes a series of maturation steps (14). Transcription factor IIIC (TFIIIC) first recognizes and binds to the internal control elements, the A- and B-boxes, within tRNA genes, facilitating the assembly of TFIIIB upstream of the transcription start site. TFIIIB then recruits RNA polymerase III, which carries out the transcription of tRNA genes. When the cellular demand for tRNA decreases, Maf1, a general repressor of RNA polymerase III transcription, directly interacts with RNA polymerase III, preventing its recruitment to TFIIIB-bound tRNA genes and thereby repressing tRNA transcription (15).

Pre-tRNA are synthesized with 5’ leader and 3’ trailer sequences, and a small fraction contains an intron. The initial processing of pre-tRNA occurs in the nucleus and begins with intron removal through a two-step process of cleavage and ligation. The cleavage is mediated by the tRNA splicing endonuclease complex in collaboration with RNA kinase Clp1, producing two exons and one intron (16). Exon ligation is subsequently catalyzed by the tRNA ligase complex (tRNA-LC), a pentameric assembly essential for generating mature tRNA (17). Following intron removal, the 5’ leader and 3’ trailer sequences are excised, and a CCA trinucleotide is enzymatically added to the 3’ end. Mature tRNA are then exported to the cytoplasm, where they perform their canonical role in translation (18).

Mature tRNA are critical adapters that decode codons on messenger RNA (mRNA) by delivering corresponding amino acids to the ribosome (19). Typically comprising 75–90 nucleotides, tRNA adopt a conserved cloverleaf-like secondary structure and an “L”-shaped tertiary conformation, maintained through extensive base pairing and stacking interactions (20–23). Structurally, tRNA consist of four major regions: the amino acid acceptor stem, the D-stem loop, the anticodon stem loop, and the T-stem loop. Each tRNA is aminoacylated by its cognate aminoacyl-tRNA synthetase (ARS), ensuring high fidelity in translating the genetic code (24–26). ARS are housekeeping enzymes that catalyze the covalent attachment of amino acids to their cognate tRNA - a pivotal step in protein biosynthesis (27). In higher eukaryotes, eight ARSs and three ARS-interacting multifunctional proteins (AIMPs) assemble into a large multi-tRNA synthetase complex, which not only facilitates translation but also regulates cellular homeostasis (28). Within the multi-tRNA synthetase complex, AIMPs primarily serve as scaffolding proteins to maintain the structural integrity and regulatory interactions of the complex (29).

Perturbations in tRNA biogenesis and metabolism can lead to mistranslation and cellular dysfunction, and are increasingly associated with neurological disorders (30). However, despite growing recognition of their significance, detailed knowledge regarding tRNA expression patterns and regulatory mechanisms within neurological disorders remains limited.

### 2.2 Stress-responsive tRNA fragmentation: biogenesis and classification of tRNA-derived small RNAs

Pre-tRNA or mature tRNA can be enzymatically cleaved to produce a variety of functional tRNA-derived small RNAs (tsRNAs) (31). These fragments have emerged as critical post-transcriptional regulators that influence gene expression under both physiological and pathological conditions. Under normal cellular conditions, tsRNAs are involved in diverse biological processes, including transcriptional repression, post-transcriptional regulation, mRNA stability regulation, and maintenance of cellular homeostasis (32, 33). tRNA share several similarities with microRNA and can influence mRNA stability by interacting with RNA-binding proteins such as the Argonaute protein family or by modulating the activity of silencing complexes that bind to the 3’ untranslated regions of target genes (34). In addition, tRNA-derived RNA fragments (tRFs) can regulate translation by modulating ribosome biogenesis. For instance, LeuCAG 3’ tsRNA can base-pair with ribosomal protein mRNA, thereby altering their secondary structures to enhance translation efficiency and promote ribosome biogenesis (32). tRFs can also modulate translation by disrupting the assembly of the translation initiation complex. They interact with eukaryotic translation initiation factor 4G (eIF4G), eIF4A, or the eIF4G/eIF4A complex, thereby suppressing translation initiation (34). In contrast, during pathological states such as autoimmune disorders, viral infections, hypoxia, cancer, ultraviolet radiation, and heat shock, the expression patterns of tsRNAs become markedly dysregulated (5, 35, 36). This dysregulation often reflects underlying cellular stress and disease-specific mechanisms, positioning tsRNAs as promising candidates for both diagnostic and therapeutic applications. For instance, tRF-20-M0NK5Y93 directly interacts with specific binding sites on the oncogenic long non-coding RNA MALAT1, a key prognostic indicator associated with tumor metastasis. Acting in a microRNA-like manner, tRF-20-M0NK5Y93 suppresses MALAT1 expression, thereby attenuating metastatic potential in colorectal cancer cells. This inhibitory effect is mediated, at least in part, through the regulation of SMC1A, linking tRFs-dependent post-transcriptional control to pathological conditions (36).

**Figure 1:**
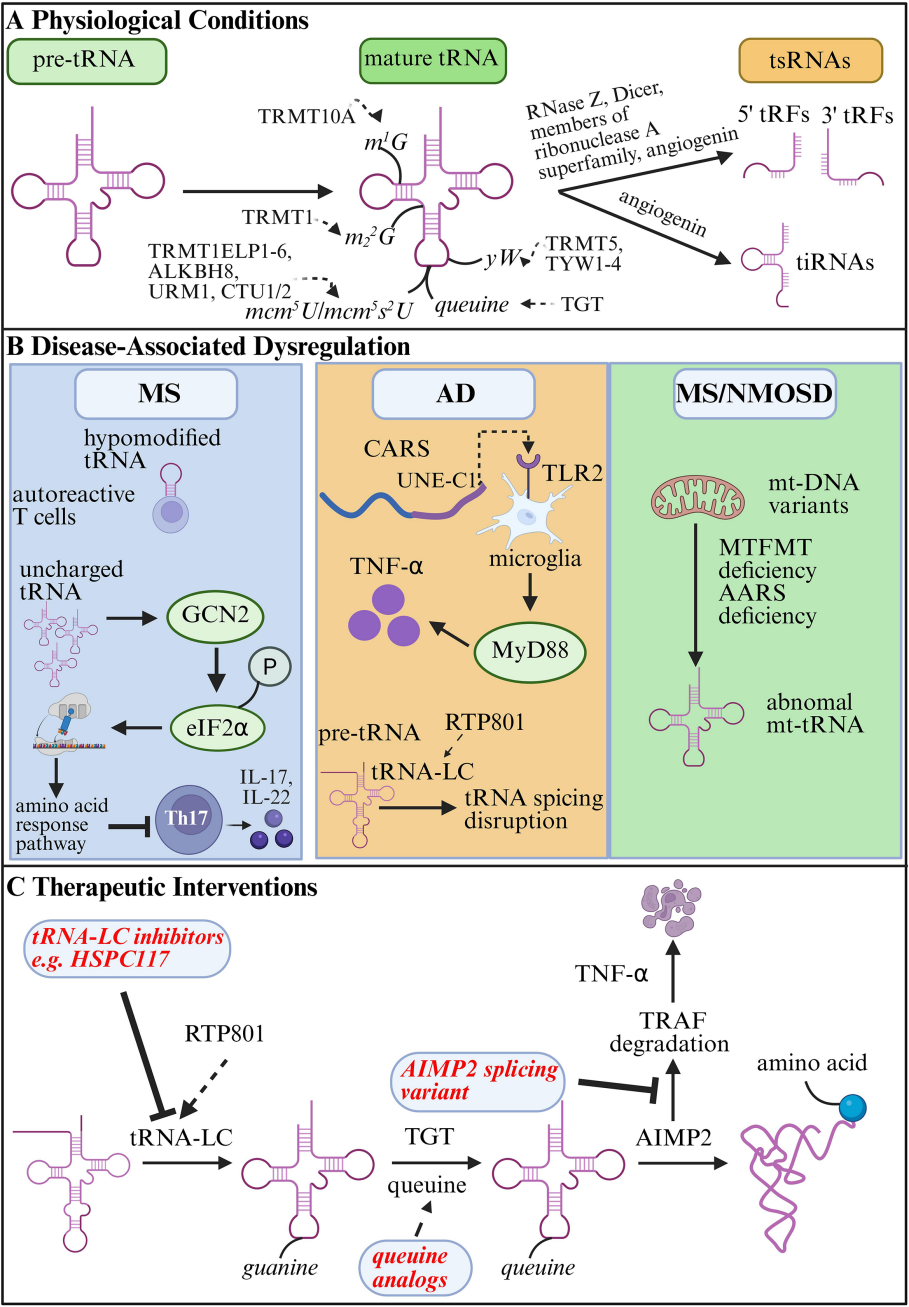
tRNA dynamics in neuroimmune homeostasis and dysregulation. **(A)** Physiological Conditions: Illustration of tRNA modifications (e.g., *queuine* modification) and fragmentation into tsRNAs under homeostatic conditions, contributing to immune tolerance and neuronal health. **(B)** Dysregulation in Neuroimmune Disorders: Hypomodified tRNA in autoreactive T cells and uncharged tRNA inhibiting the differentiation of Th17 cells (e.g., in MS); non-classical mechanisms of CARS and aberrant tRNA splicing (e.g., in AD); mt-tRNA variants and mutations in related enzymes mimicking demyelination (e.g., in NMOSD and MS). **(C)** Therapeutic Interventions: Potential therapeutic strategies targeting tRNA-related pathways, including queuine analogs, tRNA-LC inhibitors, and splice variants of AIMP2. tRNA, transfer RNA; pre-tRNA, precursor molecule; tsRNAs, tRNA-derived small RNAs; tRFs, tRNA-derived RNA fragments; tiRNAs, tRNA halves/stress-induced tRNA-derived RNAs; TGT, tRNA-guanine transglycosylase; yW, wybutosine; TRMT5, tRNA methyltransferase 5; TYW1-4, tRNA wybutosine-synthesizing enzyme 1-4; mcm5U, 5-methoxycarbonylmethyluridine; mcm5s2U, 5-methoxycarbonylmethyl-2-thiouridine; ELP1-6, the Elongator complex; ALKBH8, AlkB homolog 8 tRNA methyltransferase; URM1, ubiquitin-related modifier 1; CTU1/2, cytosolic thiouridylase subunit 1/2; m1G, 1-methylguanosine; TRMT10A, tRNA methyltransferase 10A; m22G, N2N2-dimethylguanosine; TRMT1, tRNA methyltransferase 1; MS, multiple sclerosis; GCN2, general control nonderepressible 2; eIF2α, eukaryotic translation initiation factor 2α; Th17, T helper 17 cells; IL-17, Interleukin-17; AD, Alzheimer’s disease; CARS, cytoplasmic cysteinyl-tRNA synthetase; UNE-C1, unique N-terminal extension C1 domain; TLR2, Toll-like receptor 2; MyD88, myeloid differentiation primary response 88; TNF-α, tumor necrosis factor-alpha; RTP801/REDD1, regulated in development and DNA damage responses 1; tRNA-LC, tRNA ligase complex; NMOSD, neuromyelitis optica spectrum disorder; mt-tRNA, mitochondrial tRNA; MTFMT, methionyl-tRNA formyltransferase; DARS, aspartyl-tRNA synthetase; HSPC117, an antagonist of tRNA-LC components; AIMP2, aminoacyl-tRNA synthetase-interacting multifunctional protein 2; TRAF, tumor necrosis factor receptor-associated factor.

tsRNAs are broadly classified into two major categories: tRFs and tRNA halves/stress-induced tRNA-derived RNAs (tiRNAs). Pre-tRNA or mature tRNA are cleaved at the 5’ end, 3’ end or internal sequences by RNase Z, Dicer, members of ribonuclease A superfamily, or angiogenin to generate 5’-tRFs, 3’-tRFs, and i-tRFs (37). Exposure to external stressors such as sodium arsenite, heat shock, or ultraviolet irradiation activates angiogenin, a secreted ribonuclease, which cleaves tRNA at the anticodon loop, producing tiRNAs approximately 30–45 nucleotides in length (38, 39) (**Figure 1A**). Under hypoxic conditions, Dicer1 expression is upregulated, which in turn enhances the migration and invasion of colorectal cancer cells by promoting the biogenesis and activity of tRF-20-MEJB5Y13 (40). In neuroimmune disorders, although alterations in tsRNAs have been reported, the enzymatic mechanisms underlying these changes are still under investigation.

### 2.3 Post-transcriptional modifications: guardians of tRNA stability and decoding fidelity

Beyond their canonical structure, tRNA undergo extensive post-transcriptional modifications that are crucial for maintaining their structural integrity, functional stability, and translational fidelity. These modifications, mediated by specialized enzymatic systems, promote proper codon-anticodon interactions, enhance decoding accuracy, and optimize translation efficiency (41).

In humans, tRNA exhibit more than 40 distinct types of chemical modifications, which occur at specific nucleotide positions, such as *queuine*, wybutosine (*yW*), 5-methoxycarbonylmethyluridine (*mcm^5^U*)/5-methoxycarbonylmethyl-2-thiouridine (*mcm^5^s^2^U*), N^1^-methylguanosine (*m^1^G*) and N^2^,N^2^-dimethylguanosine (*m_2_^2^G*) (**Figure 1A**). Queuine is a hypermodified 7-deazaguanine nucleobase incorporated post-transcriptionally at the wobble position of certain tRNA (42). *Queuine* modification of tRNA occurs via a unique base-for-base exchange reaction catalyzed by eukaryotic tRNA-guanine transglycosylase (TGT), replacing guanine with queuine in the tRNA anticodon loop (43). Functionally, this modification enhances translational fidelity, supports mitochondrial function, and maintains cellular homeostasis (44, 45). tRNA methyltransferase 5 (TRMT5) and tRNA wybutosine-synthesizing enzyme 1-4 (TYW1-4) catalyze the formation of a *yW*-modified tRNA at nucleotide position 37, which supports codon recognition, enhances decoding efficiency, and maintains the translational reading frame (46). The formation of the *mcm^5^s^2^U* modification is achieved through a multistep enzymatic pathway: the Elongator complex (ELP1-6) first adds a carboxymethyl group to the uridine at position 34, producing *cm^5^U*. Subsequently, AlkB homolog 8 tRNA methyltransferase (ALKBH8) catalyzes the addition of a methoxycarbonyl group to *cm^5^U*, yielding *mcm^5^U*. Finally, ubiquitin-related modifier 1 (URM1) together with cytosolic thiouridylase subunit 1/2 (CTU1/2) transfers sulfur to the C2 position of uridine, generating *s^2^U*, which ultimately combines to form the mature *mcm^5^s^2^U* modification, promoting the reading of A-ending codons and efficient translation (47). tRNA guanine methylations (*m^1^G* and *m_2_^2^G*), catalyzed by TRMT10A and TRMT1, maintain tRNA stability, enhance translational fidelity, and promote mitochondrial homeostasis (48, 49) (**Table 1**). The functional significance of these modifications is underscored by the identification of more than 50 tRNA-modifying enzymes, mutations in which have been implicated in a variety of pathological conditions (50). Aberrations in tRNA modification processes are associated with neurodevelopmental disorders, cancer, diabetes, and mitochondrial dysfunction (51, 52).

**Table 1:** Common types of tRNA modifications.

Modification	Base	Position	Enzyme	Biological function	Reference
*queuine*	Guanine	34	TGT	Enhances translational fidelity; supports mitochondrial function; maintains cellular homeostasis	(43–45)
*yW*	Guanine	37	TRMT5, TYW1-4	Supports codon recognition; enhances decoding efficiency; maintains the translational reading frame	(46)
mcm^5^U/mcm^5^s^2^U	Uridine	34	ELP1-6, ALKBH8, URM1, CTU1/2	Enhances the reading of A-ending codons; promotes efficient translation	(47)
*m¹G*	Guanine	9	TRMT10A	Maintains tRNA stability; enhances translational fidelity; promotes mitochondrial homeostasis	(48)
*m_2_^2^G*	Guanine	26	TRMT1	Maintains tRNA stability; enhances translational fidelity; promotes mitochondrial homeostasis	(49)

tRNA, transfer RNA; TGT, tRNA-guanine transglycosylase; *yW*, wybutosine; TRMT5, tRNA methyltransferase 5; TYW1-4, tRNA wybutosine-synthesizing enzyme 1-4; *mcm^5^U*, 5-methoxycarbonylmethyluridine*; mcm^5^s^2^U*, 5-methoxycarbonylmethyl-2-thiouridine; ELP1-6, the Elongator complex ; ALKBH8, AlkB homolog 8 tRNA methyltransferase; URM1, ubiquitin-related modifier 1 ; CTU1/2, cytosolic thiouridylase subunit ½; *m^1^G*, 1-methylguanosine; m_2_^2^G, N^2^,N^2^-dimethylguanosine.

A recent study demonstrated that whole-body knockout of a 2’-O-methyltransferase responsible for tRNA modification selectively triggered degradation of tRNA^Phe^ in mice. This loss of tRNA stability resulted in a specific reduction in translation efficiency at phenylalanine codons within the brain (53, 54). Interestingly, this effect was confined to the brain and not observed in other tissues, suggesting that neural tissues are particularly vulnerable to disruptions in tRNA modification.

## 3 Dysregulated tRNA dynamics in neuroimmune disorders

### 3.1 MS: tRNA modifications and tsRNAs as immunomodulatory targets

#### 3.1.1 Queuine analogs restore translational fidelity in autoactive T cells

While *queuine*-modified tRNA are abundant in terminally differentiated cells, they are often hypomodified in rapidly proliferating or activated cells, such as activated immune T cells during autoimmune responses (55) (**Figure 1B**). Given that MS is characterized by the infiltration of autoreactive T cells into the CNS, it has been hypothesized that modulating tRNA modifications specifically in these hyperactive immune cells may allow for selective immunoregulation without broadly affecting non-dividing cells (56). In experimental autoimmune encephalomyelitis (EAE), a murine model of MS, administration of a synthetic TGT substrate (*NPPDAG*) led to complete clinical remission, coupled with a marked reduction in immune activation and neurodegeneration markers within five days. This therapeutic effect, however, required functional TGT; mice lacking TGT activity failed to respond to treatment (57).

Subsequent optimization of queuine analogs revealed that only a small subset of structural variants retained or enhanced immunomodulatory efficacy, despite many being competent TGT substrates. A key structural determinant was a flexible alkyl chain of defined length, which appeared essential for activity (58). Furthermore, recognizing that queuine analogs might also influence non-immune cell types, researchers screened these compounds in rheumatoid arthritis synovial fibroblasts by measuring IL-6 secretion, followed by validation in EAE models. This approach led to the identification of two promising queuine analogs: one featuring a rigidified modification of the original structure, and the other a structurally simplified molecule incorporating an oxime motif (9).

Importantly, *queuine* is the only known RNA modification that is acquired exogenously—obtained through the diet and the gut microbiome (42). The TGT enzyme demonstrates broad substrate tolerance for structural analogs of natural queuine, raising the possibility of pharmacologically modifying tRNA function via queuine analogs (59). These findings offer a new therapeutic avenue for modulating immune function and promoting CNS repair through targeted manipulation of tRNA modification machinery. However, the precise cellular and molecular mechanisms by which queuine analogs restore immune tolerance or neuroprotection in MS remain to be fully elucidated.

#### 3.1.2 Abnormal tRNA modifications lead to translational defects

tRNA modifications are dynamically regulated during oligodendrocyte (OL) maturation. A study profiling the tRNA transcriptome during OL differentiation revealed that hypomodification of specific tRNA within or near the anticodon region, such as *mcm^5^U/mcm^5^s^2^U* and *yW*, was observed in mature OL compared to oligodendrocyte precursor cell (OPC). These modifications correlate with altered tRNA decoding capacity and mRNA stability, suggesting that tRNA modification-mediated translational control may contribute to myelination and white matter integrity (60).

Moreover, genetic perturbations in tRNA modification enzymes further highlight the role of tRNA modifications in CNS and immune dysfunction. tRNA-modifying enzymes are indispensable for tRNA modifications and for maintaining tRNA structure and function. Among them, TRMT10A catalyzes the *m^1^G* modification of several cytoplasmic tRNA, which is essential for preserving the steady-state abundance of multiple *m^1^G*-containing species (61). This modification plays a particularly important role in sustaining efficient translation of neuron-associated mRNA. Loss of TRMT10A causes a broad reduction in tRNA^iMet^ and tRNA^Gln(CUG)^ levels, leading to translational defects and disrupted protein synthesis. Knockout of TRMT10A results in impaired brain function in mice (62). Similarly, TRMT1, which catalyzes the *m_2_^2^G* modification, is essential for both mitochondrial and cytosolic tRNA integrity. TRMT1 deficiency in zebrafish leads to immune imbalance and neurodegeneration (63).

#### 3.1.3 tsRNAs signatures in biofluids: toward non-invasive diagnosis

Most MS patients (approximately 85%) initially present with the relapsing-remitting form (RRMS), which may later evolve into secondary progressive MS (SPMS) (64). Early diagnosis and stratification are critical, as timely therapeutic intervention can significantly delay disease progression (65). A recent study has demonstrated that tRFs can be packaged into exosomes and released into extracellular fluids, including plasma and cerebrospinal fluid (CSF), making them detectable (66).

Integrated small RNA profiling has enabled the differentiation of RRMS and SPMS patients from neurological disease controls. Notably, tRF-36-PJB7MNLE308HP1B emerged as the top discriminative feature, being upregulated specifically in RRMS patients. Interestingly, no significant differences in tRFs expression were observed between MS patients in relapse versus remission phases or between inflammatory and non-inflammatory disease controls. This suggests that tRFs signatures may be MS-specific and stable, rather than merely reflecting transient inflammation (67). These findings support the potential of tsRNAs as reliable biomarkers for early MS diagnosis. However, since the levels of tsRNAs do not fluctuate with changes in the inflammatory state of MS, they cannot be used to monitor disease progression.

In recent years, the prominence of tRFs in body fluids has sparked interest in their utility as biomarkers for liquid biopsy in various human diseases, including neurological disorders (68). Three specific tRFs (5’AlaTGC, 5’GlyGCC, and 5’GluCTC) have been detected in the plasma of pre-epileptic patients prior to the onset of overt seizure (69). A highly sensitive and specific detection platform enables the quantification of these tRFs from minimal plasma volumes using standard benchtop equipment (70). This finding highlights the promise of tRFs as early-warning biomarkers of neuronal hyperexcitability. While such tRFs may not directly mirror immune activation, they illustrate the broader concept that extracellular tRFs signatures can capture early pathological alterations across diverse CNS disorders. However, their application in clinical settings remains at a preliminary stage, and no tRFs-based diagnostic tools have yet been implemented in routine practice.

#### 3.1.4 tRNA and its derivatives in the pathogenesis of MS

Recent evidence suggests that tRNA and its derivatives dysregulation contributes to multiple pathological processes in MS, ranging from immune activation to impaired myelin repair. For instance, the accumulation of uncharged tRNA can engage and activate the protein kinase general control nonderepressible 2 (GCN2). Once activated, GCN2 undergoes autophosphorylation and phosphorylates the translation initiation factor eIF2α, a pivotal regulator of the integrated stress response. Phosphorylated eIF2α transiently suppresses global cap-dependent translation, while promoting the selective translation of specific mRNA that initiate the amino acid response pathway (**Figure 1B**). Despite these insights, the precise mechanisms by which activation of the amino acid response pathway inhibits T helper 17 cell differentiation and consequently attenuates EAE progression remain unresolved (71, 72). Meanwhile, tumor necrosis factor-alpha (TNF-α), a central mediator of neuroinflammation in MS, has been linked to several tsRNAs (73). tRFs such as tRF-Ser-GCT-113, tiRNA-His-GTG-001, and tRF-Gln-TTG-035 may modulate the TNF signaling pathway (74). While 5’tiRNA^Gly^ has been reported to inhibit TNF-α expression (75). These findings suggest that tsRNAs may contribute to the control of neuroinflammation in MS through regulating TNF signaling, although this hypothesis warrants further validation in models of MS.

In addition, tRNA^Arg^ can serve as a donor for arginyl-tRNA-protein transferase (ATE1), enabling the covalent transfer of the arginine residue to the N-terminus or specific side chains of target proteins. ATE1 has been shown to mediate post-translational arginylation of β-actin, influencing cytoskeletal organization critical for OPC migration and myelination (76, 77). ATE1 expression peaks during myelination, and its downregulation impairs OL differentiation, reduces myelin thickness, and leads to motor deficits in mice (78, 79). These findings imply that tRNA-mediated arginylation is essential for remyelination and CNS repair in demyelinating diseases such as MS.

Together, these findings highlight the central role of tRNA-associated enzymes, tsRNAs, and tRNA modifications in maintaining neuroimmune balance and myelination, and suggest that targeting these pathways may provide novel strategies for both neuroprotection and immune regulation in MS.

### 3.2 NMOSD and ALS: AIMPs as neuroimmune biomarkers and neuroinflammatory modulators

AIMP1, also known as p43, is a core structural component of the multi-ARS complex. Recent evidence suggests that AIMP1 plays a role as a biomarker in NMOSD. Plasma AIMP1 levels were found to be elevated in patients with acute aquaporin-4 antibody-positive NMOSD (AQP4-IgG+ NMOSD) compared to both healthy controls and those in remission. In addition, plasma AIMP1 levels were significantly higher in patients with moderate to severe NMOSD compared to those with mild NMOSD and healthy controls. AIMP1 levels were identified as an independent predictor for the risk of developing moderate to severe NMOSD. The optimal threshold for predicting moderate to severe disease was determined to be 49.55 pg/mL. Treatment with intravenous *methylprednisolone* significantly lowered AIMP1 concentrations. Therefore, plasma AIMP1 may serve as a promising biomarker for assessing NMOSD severity, as well as a valuable dynamic indicator of disease activity and therapeutic response in acute AQP4-IgG+ NMOSD (10). However, the current findings are derived from a limited cohort in northern China. The study was limited by its relatively small sample size of fewer than 100 participants drawn from a single region, which restricts both statistical power and generalizability. Furthermore, for protein biomarkers such as AIMP1, assay standardization across laboratories and longitudinal sampling remain insufficient. These limitations highlight that the validation through larger, multicenter studies across diverse populations is essential. Moreover, further investigation into the mechanistic role of AIMP1 in the pathogenesis of NMOSD is warranted.

Immune cell infiltration and immune dysregulation are increasingly recognized as contributing factors in ALS pathogenesis (80, 81). AIMP2, typically released from the multi-tRNA synthetase complex, is implicated in TNF-α-induced cell death via degradation. However, overexpression of an exon 2-deleted splicing variant of AIMP2 was found to antagonize this effect. This variant competes with full-length AIMP2, preventing tumor necrosis factor receptor-associated factor 2 (TRAF2) degradation and downregulating neuroinflammatory pathways. In mouse models, this intervention delayed ALS symptom onset and extended survival (82). Therefore, AIMP2 variants exhibit the potential to modulate neuroimmune responses, thereby representing promising therapeutic targets in ALS.

### 3.3 AD and PD: tRNA dysregulation and neuroimmune perturbation

#### 3.3.1 Non-classical mechanisms of ARS and tRNA splicing impairment in AD

AD is an age-related neurodegenerative disorder characterized by the accumulation of β-amyloid (Aβ) plaques and progressive neuronal loss (83). Immunotherapy studies have demonstrated that microglia are recruited to Aβ plaques, contributing to their clearance, thereby highlighting the importance of immune processes in both AD pathogenesis and therapeutic response (84).

Cytoplasmic cysteinyl-tRNA synthetase (CARS), a member of the ARS family, catalyzes the ligation of cysteine to tRNA^cys^ (85). Human CARS contains several additional domains, including unique N-terminal extension C1 domain (UNE-C1), which functions as an endogenous ligand for Toll-like receptor 2 (TLR2) (86). This enables CARS to play immune-related roles beyond aminoacylation. Microglia, which uniquely express nearly all known TLRs in the CNS, are activated in transgenic mouse models of AD by neuronal overexpression of CARS (87). This activation triggers the TLR2/myeloid differentiation primary response 88 (MyD88) pathway and leads to increased TNF-α expression (**Figure 1B**). TNF-α stimulation of SH-SY5Y neuroblastoma cells, in turn, upregulates CARS expression and secretion. *In vitro*, CARS treatment also promotes microglial chemotaxis and pro-inflammatory cytokine production through TLR2/MyD88 signaling (88). However, key questions remain, including the mechanisms of CARS secretion from neurons, how pro-inflammatory factors regulate the expression of CARS in neurons and whether CARS targeted knockout can ameliorate AD pathology.

Additionally, the stress-responsive protein regulated in development and DNA damage responses 1 (RTP801/REDD1) has been shown to interact with the tRNA-LC, disrupting tRNA splicing and impairing X-box binding protein 1 (XBP1)-mediated unfolded protein response. This interaction contributes to neuroinflammation and cognitive decline in AD (**Figure 1B**). Elevated RTP801 levels result in intron-containing pre-tRNA accumulation and defective XBP1 splicing, ultimately impairing neuronal function (89).

Another tRNA-related mechanism involves tyrosyl-tRNA synthetase (TyrRS). *Resveratrol* has been shown to activate TyrRS, which in turn triggers downstream PARP1 and SIRT1 signaling pathways, promoting autophagy and mitigating Aβ25-35-induced neurotoxicity in PC12 cells (90).

These findings suggest that ARSs, including CARS and TyrRS, and tRNA-LC are important mediators linking tRNA biology to neuroimmune responses in AD.

#### 3.3.2 tRFs and inflammasome regulation in PD

Neuroinflammation is a well-recognized feature of PD, supported by findings from postmortem brain tissue, clinical biological samples analyses, and both *in vitro* and *in vivo* models (91). Microglia, particularly abundant in the substantia nigra and striatum, are key drivers of PD-associated neuroinflammation (92). Among immune signaling components, the NLRP3 inflammasome plays a central role in PD neuroinflammation (93). A recent study identified tRF-02514 as a potential therapeutic target. In a PD mouse model induced by 1-methyl-4-phenyl-1,2,3,6-tetrahydropyridine, inhibition of tRF-02514 reduced the activation of the NLRP3 inflammasome, thereby decreasing pyroptosis and pro-inflammatory cytokine release. Concurrently, it promoted autophagy by upregulating ATG5, enhancing the clearance of toxic substances and maintaining cellular homeostasis. These findings suggest that tRF-02514 regulates both inflammatory and metabolic pathways, supporting its role in neuronal survival and therapeutic potential in PD (13).

Beyond tRF-02514, patients with PD exhibit elevated levels of tRFs containing a conserved RGTTCRA motif and reduced level of mitochondrial-derived tRFs (mt-tRFs) in the substantia nigra, cerebrospinal fluid, and blood (94). These motif-specific alterations indicate that the tRFs landscape in PD biofluids is more diverse than previously recognized. Whether this motif enrichment is mechanistically connected to immune signaling pathways or reflects neuronal stress responses remains to be determined.

### 3.4 Nervous system injury and infection: tRFs as dynamic modulators of neuroimmune responses

Emerging evidence indicates that tRFs play a crucial role in modulating immune responses following CNS injury in ischemic stroke. Transcriptomic analyses of peripheral blood from stroke patients revealed a global downregulation of microRNA coupled with a significant upregulation of specific tRFs, suggesting a dynamic reprogramming of small RNA-mediated regulatory networks in the post-stroke period (95). This shift in small RNA expression coincides with activation of the cholinergic anti-inflammatory reflex, a key neuroimmune pathway that dampens peripheral immune responses following CNS injury (96). Among immune cells, CD14^+^ monocytes have been identified as central mediators in this regulatory process, displaying robust expression of stroke-induced tRFs. Functional studies demonstrated that these tRFs act in a microRNA-like fashion to modulate immune gene expression. For instance, overexpression of tRF-22-WE8SPOX52 suppresses the expression of Zbp1, a damage-associated molecular pattern sensor involved in interferon signaling and inflammasome activation (97). Moreover, the expression of several tRFs is dynamically regulated by inflammatory stimuli such as lipopolysaccharide, and is modifiable by immunosuppressive agents including nicotine and dexamethasone. These findings suggest that tRFs serve as rapid responders to immune stress, actively contributing to the balance between inflammation and immunosuppression during the acute and subacute phases of ischemic stroke (95).

Beyond ischemic stroke, tRFs have been implicated in glial activation and inflammatory cytokine regulation in a range of CNS conditions. In the retina, Müller cells, specialized radial glia that support retinal architecture and function, are important mediators of retinal inflammation. The tsRNA Gln-i-0095 has been shown to function as a dual-gene regulator, silencing NFIA and TGFBR2 through a microRNA-like mechanism. This regulation induces reactive gliosis in Müller cells, leading to the release of pro-inflammatory cytokines, including IL-1β, IL-6, and TNF-α. This response exacerbates retinal ganglion cell damage via reactive oxygen species and downstream cytokine effects, suggesting a pathogenic role in ischemic retinopathy (98). Similarly, tRF-41 has been found to promote the expression of IL-1 and IL-6 in human astrocytes, thereby contributing to neuroinflammation in spinal cord injury models (99). In retinitis pigmentosa, a hereditary neurodegenerative condition marked by photoreceptor apoptosis and immune dysregulation, tRFs may contribute to immune homeostasis (100). The tRF Other-1-17-tRNA-Phe-GAA-1-M3 has been reported to alleviate microglia-mediated neuroinflammation by upregulating SRC mRNA expression and promoting the transition of disease-associated microglia to a homeostatic state in rat models (86). This highlights the potential of tRFs to modulate immune cell phenotypes in neurodegenerative retinal disease.

Flaviviruses such as Japanese encephalitis virus (JEV) and West Nile virus (WNV) are major pathogens responsible for viral encephalitis and CNS injury in humans. JEV remains the leading cause of mosquito-borne encephalitis worldwide, while WNV is the predominant agent of epidemic encephalitis in North America (101, 102). Recent transcriptomic investigations have shown that flavivirus infection is associated with the upregulation of multiple tRNA synthetases in the brain. These enzymes may participate in the neuroinflammatory responses induced by flaviviral replication and host immune activation, although their exact contributions remain to be fully elucidated (103). The altered expression of tRNA synthetases and potentially dysregulated tRNA charging may reflect a broader disturbance of RNA metabolism and protein homeostasis during viral encephalitis, thereby contributing to neuronal injury and immune dysfunction. Consistent with this notion, the emergence of tRF-5’ LysTTT was shown to promote the survival of cholinergic neurons upon exposure to botulinum neurotoxins. Mechanistically, tRF-5’LysTTT forms a complex with the RNA-binding protein HNRNPM, modulating its expression levels and thereby suppressing ferroptosis (104). This finding highlights how exogenous toxins can disrupt RNA metabolism and induce tRNA cleavage, likely through elevated expression or activity of specific nucleases. Although the direct neuroimmune implications remain unclear, these observations suggest that enzymatic regulation of tRFs biogenesis may represent a critical intersection between antimicrobial defense and neuroimmune dysregulation. Further research is needed to define the roles of tRNA-related pathways in viral neuropathogenesis, including the involvement of specific tRNA modifications, tsRNAs, or ARS activities in regulating antiviral immunity and CNS inflammation.

## 4 Mitochondrial tRNA variants: overlooked players in neuroimmune pathogenesis

Mitochondrial dysfunction is increasingly recognized as a contributing factor in autoimmune neurological disorders such as MS and NMOSD (105, 106). Furthermore, mutations in mitochondrial tRNA (mt-tRNA) genes are believed to contribute to disease via disruptions in mitochondrial transcription and translation (107). However, there is limited research on the role of mutations or dysregulation of mt-tRNA in neuroimmune diseases, and existing studies are controversial.

The human mitochondrial genome encodes a distinct set of 22 mt-tRNA. In contrast to their nuclear counterparts, mt-tRNA are characterized by less stable structures and a high reliance on protein interactions, due to their A/U-rich sequences, predominantly noncanonical tertiary conformations and reduced level of modification (108, 109). Moreover, mt-tRNA exhibit sufficient sequence divergence from nuclear tRNA, allowing clear distinction between nuclear tRNA and mt-tRNA on sequence identity. Notably, nuclear-encoded lookalikes of mt-tRNA also exist, though their biological significance remains uncertain (110). In addition, mt-tRNA display broad tissue distribution, with abundance varying across tissues, and are often more prevalent than nuclear tRNA in cancer (111). Taken together, these features underscore that mt-tRNA are not simply by-products of mitochondrial RNA turnover but instead constitute a distinct dimension of small RNA biology with potential immunological and pathological significance.

Specific mitochondrial DNA variants such as G15257A and G15812A in the tRNA^Thr^ gene have been linked to MS susceptibility in German (n = 100) and American (n = 53) populations, however, this association has not been confirmed in Iranian population (n = 100) (112–114). All three studies employed a case-control design, in which peripheral blood samples were collected from patients for DNA extraction and analysis. The observed discrepancies may stem from differences in population genetics or diagnostic criteria. Alternatively mt-tRNA variants may function as genetic modifiers rather than primary risk alleles, with their impact becoming evident only in the context of environmental exposures or additional nuclear variants. Hence, the role of mt-tRNA variants in neuroimmune disorders remains unresolved, and rigorous multicenter studies integrating both nuclear and mitochondrial genetics will be needed to clarify causality.

Mutations in the mitochondrial methionyl-tRNA formyltransferase (MTFMT) gene have been associated with neuroimaging features resembling acquired demyelinating diseases such as MS and NMOSD (115). Additionally, deficiency of mitochondrial aspartyl-tRNA synthetase (DARS) leads to a distinct form of leukoencephalopathy, characterized by spinal cord and brainstem involvement, lactic acidosis, and white matter changes on MRI—features that may mimic MS or other inflammatory demyelinating disorders, especially in cases with a relapsing course and steroid responsiveness (116–118). Similarly, one study identified a mutation in the mt-tRNA^Ile^ gene in a patient presenting with chronic progressive external ophthalmoplegia and clinical features resembling MS (119). These cases highlight the diagnostic challenges in differentiating mitochondrial leukoencephalopathies from classic neuroimmune disorders and underscore the need for careful genetic and metabolic evaluation (**Figure 1B**).

To date, research on mt-tRNA in neuroimmune diseases remains largely exploratory. While these findings reveal intriguing overlaps between mitochondrial dysfunction and immune-mediated demyelination, no mt-tRNA-based biomarkers have yet been validated for clinical use. Although mt-tRNA mutations can mimic acquired demyelinating disorders, direct causal evidence linking mt-tRNA mutations to neuroimmune disorders is still lacking. Nevertheless, several investigations on mt-tRNA dysregulation in other pathological contexts may provide valuable mechanistic insights and serve as conceptual frameworks to guide future research in neuroimmune disorders. For instance, recent evidence has identified DARS2 as the first mitochondrial tRNA synthetase with a defined role in modulating host immune responses. In bacterial pneumonia, DARS2 is released into the circulation, where it contributes to innate immune activation and tissue repair. Mechanistically, DARS2 associates with the bacterially induced ubiquitin E3 ligase subunit FBXO24, which mediates its ubiquitination and proteasomal degradation. This process is negatively regulated by acetylation of DARS2, which stabilizes the enzyme. In experimental models of pneumonia, Fbxo24-deficient mice show elevated DARS2 levels accompanied by enhanced pulmonary immune cell infiltration and cytokine production, indicating that DARS2 accumulation amplifies host immune responses (120). In AD brains, cholinergic-targeting mt-tRFs decline preferentially in females, indicating a sex-specific vulnerability (8). Consistently, analyses of neonatal umbilical cord blood serum have revealed sex-dependent alterations in mt-tRFs landscapes, with female newborns showing more pronounced shifts in response to maternal stress (121). Collectively, sex-specific differences are also a critical dimension in understanding mt-tRFs biology across neuroimmune and neurodegenerative contexts.

## 5 tRNA-centric innovations in diagnostics and therapeutics

### 5.1 Liquid biopsy platforms: tsRNAs and AIMP1 as clinical biomarkers

Liquid biopsy has emerged as a transformative tool for non-invasive disease monitoring, particularly in neuroimmune disorders. tsRNAs, including tRFs and tiRNAs, are stable in biofluids such as plasma and CSF, making them ideal candidates for biomarker discovery. For instance, in MS, tRF-36-PJB7MNLE308HP1B is upregulated in RRMS patients and distinguishes MS subtypes from other neurological conditions, suggesting its utility in early diagnosis (67). Unlike traditional inflammatory markers, tsRNAs signatures remain stable across disease phases, offering MS-specific diagnostic potential. However, their inability to reflect dynamic inflammatory changes limits their use in tracking progression. AIMP1 may serve as a potential severity biomarker in AQP4-IgG+ NMOSD. Its plasma levels correlate with disease activity and decline after corticosteroid therapy, indicating a dual role in diagnosis and treatment response (10).

Despite these advances, clinical implementation remains nascent. Challenges include standardizing detection methods (e.g., small RNA sequencing for tsRNAs, ELISA for AIMP1) and validating findings across diverse cohorts. Future efforts should integrate multi-omics platforms to enhance biomarker specificity and explore combinatorial panels (e.g., tsRNAs + AIMP1) for improved stratification of neuroimmune disorders (**Table 2**).

**Table 2:** tRNA-derived biomarkers in neuroimmune disorders.

Biomarker	Disease	Object	Biofluid	Diagnostic utility	Reference
tRF-36-PJB7MNLE308HP1B	MS	Human	Plasma, CSF	Distinguishes RRMS from SPMS and non-inflammatory controls; stable across disease phases	(67)
AIMP1	NMOSD	Human	Plasma	Predicts acute severity (threshold: 49.55 pg/mL); correlates with steroid response	(10)
tRF-02514	PD	Mouse	Extracellular vesicles	Suppresses NLRP3 inflammasome; promotes autophagy and neuronal survival	(13)
tsRNA-Gln-i-0095	Ischemic Retinopathy	Mouse	Retinal tissue	Induces reactive gliosis; therapeutic inhibition reduces inflammation	(98)

tRNA, transfer RNA; tRFs, tRNA-derived RNA fragments; tsRNAs, tRNA-derived small RNAs; MS, multiple sclerosis ; CSF, cerebrospinal fluid; RRMS, relapsing-remitting multiple sclerosis; SPMS, secondary progressive multiple sclerosis; AIMP1, aminoacyl-tRNA synthetase-interacting multifunctional protein 1; NMOSD, neuromyelitis optica spectrum disorder; PD, Parkinson’s disease; NLRP3, NOD-like receptor thermal protein domain associated protein.

### 5.2 Precision targeting of tRNA modification

#### 5.2.1 Queuine analogs: selective immunomodulation in MS

In MS, autoreactive T cells exhibit *queuine* hypomodification, contributing to immune hyperactivity. Synthetic queuine analogs restore tRNA queuine levels in EAE models, inducing rapid remission by suppressing T cell activation and neurodegeneration (57). Structural optimization of these analogs identified key features (e.g., flexible alkyl chains) necessary for efficacy, with lead compounds demonstrating reduced IL-6 secretion in rheumatoid arthritis models and improved CNS repair in EAE (9, 58).

Notably, queuine is diet- and microbiome-derived, underscoring the gut-brain-tRNA axis as a modifiable therapeutic target. However, challenges remain in achieving cell-type specificity, as systemic TGT inhibition may affect non-immune cells. Emerging strategies include prodrug formulations or nanoparticle-based delivery to target autoreactive T cells selectively. Clinical trials evaluating queuine analogs in MS are warranted to validate their translational potential.

#### 5.2.2 tRNA ligase complex antagonists: restoring proteostasis in neurodegeneration

The tRNA-LC is essential for tRNA splicing and maturation. In AD, dysregulation of tRNA-LC activity by RTP801 disrupts XBP1 splicing and the unfolded protein response, exacerbating neuroinflammation and cognitive decline (89). Antagonists of tRNA-LC components such as *HSPC117*, could restore proteostasis by preventing aberrant tRNA splicing and promoting neuronal survival. Preclinical studies highlight tRNA-LC inhibition as a dual strategy to mitigate protein aggregation and inflammasome activation. For example, in AD models, targeting RTP801-tRNA-LC interactions reduced intron-containing pre-tRNA accumulation and improved synaptic function. Similarly, in PD, modulating tRNA splicing machinery may alleviate NLRP3 inflammasome-driven neurotoxicity (13). Future research should focus on developing blood-brain barrier-penetrant inhibitors and assessing their safety in chronic neurodegeneration (**Figure 1C**).

By leveraging tsRNA stability and enzymatic vulnerabilities in tRNA modification pathways, these innovations bridge mechanistic insights into actionable clinical strategies (**Table 3**). Challenges such as biomarker validation, drug delivery, and off-target effects underscore the need for interdisciplinary collaboration to advance tRNA-centric precision medicine.

**Table 3:** tRNA-modifying enzyme targets and therapeutic candidates.

Target	Disease	Object	Therapeutic agent	Mechanism	Preclinical efficacy	Reference
TGT (*queuine* modification)	MS	Mouse	*NPPDAG* (queuine analog)	Restores tRNA queuine levels; suppresses autoreactive T cell activation	Complete remission in EAE models; reduces IL-6 secretion	(57, 58)
tRNA-LC	AD	Transgenic mouse line 5xFAD, rat primary cortical neurons, mouse primary hippocampal neurons, HEK293 cell line	*HSPC117*	Prevents aberrant tRNA splicing; restores XBP1-mediated proteostasis	Reduces pre-tRNA accumulation; improves synaptic function	(89)
AIMP2 splicing variant	ALS	HEK293 cell line	Exon 2-deleted AIMP2	Blocks TRAF2 degradation; downregulates neuroinflammatory pathways	Delays symptom onset; extends survival in mouse models	(82)

tRNA, transfer RNA; TGT, tRNA-guanine transglycosylase; MS, multiple sclerosis; *NPPDAG*, a synthetic TGT substrate; EAE, experimental autoimmune encephalomyelitis; IL-6, Interleukin-6; tRNA-LC, tRNA ligase complex; AD, Alzheimer’s disease; *HSPC117*, an antagonist of tRNA-LC components; XBP1, X-box binding protein 1; pre-tRNA, precursor tRNA; AIMP2, aminoacyl-tRNA synthetase-interacting multifunctional protein 2; ALS, amyotrophic lateral sclerosis; TRAF2, tumor necrosis factor receptor-associated factor 2.

## 6 Unresolved challenges and future directions

### 6.1 Decoding the gut-brain-tRNA axis

The gut microbiome serves as a critical source of queuine, a tRNA modification substrate incorporated via TGT to enhance translational fidelity and immune regulation (42, 44). In neuroimmune disorders like MS, *queuine* hypomodification in autoreactive T cells correlates with immune hyperactivity, and dietary or microbial queuine supplementation has shown therapeutic potential in preclinical models (57). However, key questions remain (1): Mechanistic Gaps: How do specific gut microbial taxa regulate queuine synthesis or metabolism? Are there microbiome-derived metabolites beyond queuine that influence tRNA modifications? (2) Translational Barriers: Individual variations in gut microbiota composition may affect queuine bioavailability, complicating therapeutic standardization (3). Therapeutic Opportunities: Probiotic interventions or queuine-enriched diets could modulate CNS immunity, but their efficacy and safety in humans are untested.

Future studies should integrate multi-omics (metagenomics, metabolomics, tRNA modification profiling) to map gut-tRNA-CNS crosstalk. Longitudinal cohorts could assess how microbiome shifts during disease progression impact tRNA modification landscapes, informing personalized dietary or microbial therapies.

### 6.2 Single-cell tRNAomics: resolving cell-type-specific dysregulation

Current bulk sequencing approaches obscure cell-type-specific tRNA modification dynamics, limiting insights into neuroimmune pathology. For example, OLs exhibit unique anticodon-region hypomodifications during myelination (79), while microglial tRNA modifications may regulate inflammasome activation (13). Emerging technologies like single-cell tRNA sequencing and spatial tRNA modification mapping could resolve these heterogeneities. Challenges include (1): Technical Limitations: Low abundance of tRNA molecules per cell and the lack of high-throughput methods for detecting rare modifications (e.g., *queuine*, *yW*) (2). Functional Annotation: Linking specific tRNA modifications to cell-type-specific translational programs (e.g., pro-inflammatory cytokine production in microglia vs. remyelination in OL).

Future efforts should prioritize developing CRISPR-based tools for editing tRNA modifications in specific neural or immune subsets and leveraging organoid models to study their functional consequences. Collaborations between computational biologists and chemists are essential to advance single-cell tRNAomics into a clinically actionable framework.

### 6.3 Clinical translation: from prevalidation to trials

Despite promising preclinical data, tRNA-centric diagnostics and therapeutics face significant hurdles (1): Biomarker Validation: Plasma tsRNAs (e.g., tRF-36-PJB7MNLE308HP1B in MS) and AIMP1 in NMOSD require standardization across platforms and validation in diverse, multiethnic cohorts to avoid confounding factors like age, sex, or comorbidities (10, 67) (2). Drug Delivery: queuine analogs or tRNA-LC antagonists must achieve CNS penetration without off-target effects. Nanoparticle delivery systems or Trojan horse strategies that exploit endogenous transport systems (e.g., transferrin receptor-mediated transcytosis across the blood-brain barrier) will be essential to mitigate these barriers and enhance specificity before clinical translation (122, 123) (3). Safety and Efficacy: Chronic modulation of tRNA modifications may disrupt global translation or mitochondrial function. Phase 0 trials using patient-derived induced pluripotent stem cells (iPSC) models could preclinically assess toxicity (**Table 4**).

**Table 4:** Challenges and solutions for tRNA-centric clinical translation.

Challenge	Proposed solution	Example
Biomarker validation	Multi-omics integration (e.g., tsRNAs + AIMP1 panels) and multiethnic cohort studies	Combinatorial panels for MS/NMOSD stratification
BBB penetration	Nanoparticle delivery systems or Trojan horse strategies (e.g., transferrin receptor coupling)	Queuine analogs conjugated to BBB-targeted nanoparticles
Off-target effects	Phase 0 trials using patient-derived iPSC models to assess toxicity	iPSC-derived microglia/oligodendrocytes for testing tRNA-LC antagonists

tsRNAs, tRNA-derived RNA fragments; AIMP1, aminoacyl-tRNA synthetase-interacting multifunctional protein 1; MS, multiple sclerosis; NMOSD, neuromyelitis optica spectrum disorder; BBB, blood-brain barrier; iPSC, induced pluripotent stem cells; tRNA-LC, tRNA ligase complex.

**Figure 2:**
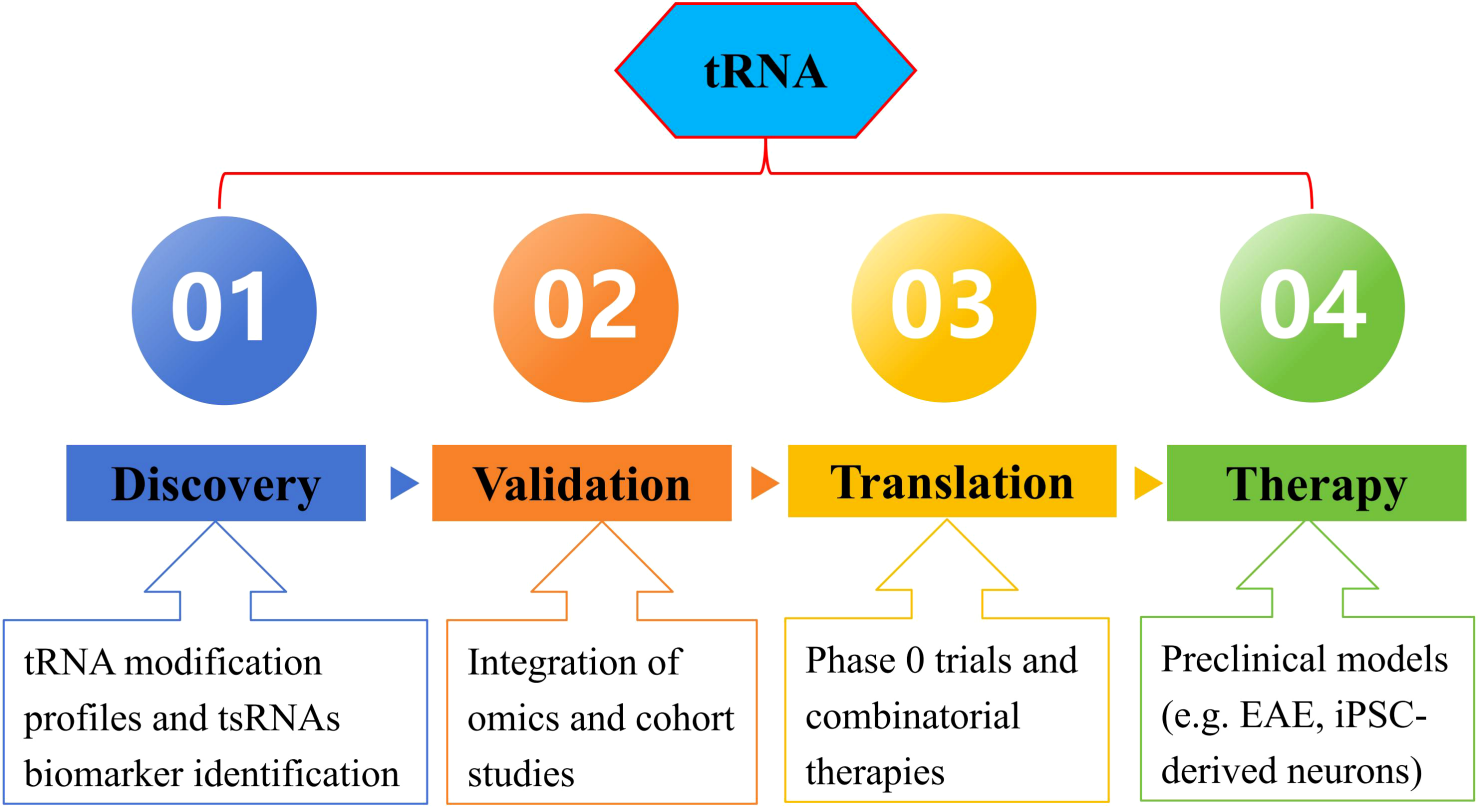
Diagnostic and therapeutic pipeline for tRNA-centric strategies. tRNA, transfer RNA; tsRNAs, tRNA-derived RNA fragments; EAE, experimental autoimmune encephalomyelitis; iPSC, induced pluripotent stem cells.

To accelerate translation, consortia should establish shared biorepositories for tRNA-related biomarker discovery and harmonize protocols for tRNA-modifying enzyme inhibitor screening. Early-phase trials in MS or NMOSD could test queuine analogs alongside immune monitoring, while neurodegenerative disease trials might combine tRNA-LC antagonists with proteostasis enhancers (**Figure 2**).

## 7 Conclusion: tRNA biology as a gateway to neuroimmune precision medicine

The evolving understanding of tRNA biology has unveiled its central role in bridging translational regulation, immune homeostasis, and neural integrity. Once viewed merely as molecular adaptors in protein synthesis, tRNA and their derivatives, such as tsRNAs and modified tRNA species, now emerge as dynamic players in neuroimmune disorders. This synthesis of mechanistic insights highlights actionable strategies to leverage tRNA-centric approaches for biomarker discovery and targeted therapies.

### 7.1 Mechanistic foundations

Dysregulated tRNA modifications (e.g., *queuine* hypomodification in MS), stress-induced tRNA fragmentation, and mt-tRNA variants disrupt neuroimmune equilibrium by impairing translational fidelity, amplifying inflammatory cascades, and compromising mitochondrial function (42, 57, 89). For instance, *queuine*-modified tRNA regulate autoreactive T cell activation in MS, while tsRNAs like tRF-02514 modulate NLRP3 inflammasome activity in PD, linking tRNA dynamics to both immune dysregulation and neuronal survival (13, 57). These findings underscore tRNA biology as a nexus for understanding disease pathogenesis and designing precision interventions.

### 7.2 Diagnostic innovations

The stability and disease specificity of tsRNAs in biofluids (e.g., plasma tRF-36-PJB7MNLE308HP1B in MS) and of secreted proteins like AIMP1 in NMOSD position them as promising non-invasive biomarkers (10, 67). Unlike transient inflammatory markers, tsRNAs signatures offer stable diagnostic profiles across disease phases, enabling early detection and subtype stratification. However, clinical adoption requires standardized detection protocols and validation in diverse populations to address variability in sample processing and comorbidities.

### 7.3 Therapeutic opportunities

Precision targeting of tRNA-modifying enzymes exemplifies the translational potential of tRNA-centric strategies. Queuine analogs restore tRNA queuine levels in autoreactive T cells, suppressing neuroinflammation in preclinical MS models (10, 57). Similarly, antagonizing aberrant tRNA splicing via tRNA ligase complex inhibitors (e.g., targeting RTP801 in AD) could alleviate proteotoxic stress and inflammasome activation (89). Challenges remain in optimizing blood-brain barrier penetration and minimizing off-target effects, yet advances in nanoparticle delivery and prodrug design hold promise for enhancing therapeutic specificity.

### 7.4 Future horizons

To fully harness tRNA biology for neuroimmune precision medicine, interdisciplinary efforts must address unresolved challenges (1): Gut-Brain-tRNA Axis: Deciphering how microbiome-derived metabolites like queuine shape CNS immunity could unlock dietary or probiotic interventions (42, 44) (2). Single-Cell tRNAomics: Resolving cell-type-specific tRNA modification landscapes will clarify their roles in microglial activation, OL differentiation, and neuronal vulnerability (13, 79) (3). Clinical Translation: Biomarker validation pipelines and phased clinical trials, guided by patient-derived iPSC models, are critical to transition tRNA-based therapies from bench to bedside.

By integrating molecular insights with technological innovation, tRNA biology offers a transformative framework for diagnosing, stratifying, and treating neuroimmune disorders. Collaborative efforts across genetics, immunology, and bioengineering will be essential to translate these discoveries into therapies that restore neuroimmune homeostasis and improve patient outcomes.

## References

[1] Hoagland MB Stephenson ML Scott JF Hecht LI Zamecnik PC . A soluble ribonucleic acid intermediate in protein synthesis. J Biol Chem. (1958) 231:241–57. doi: 10.1016/S0021-9258(19)77302-5, PMID: 13538965

[2] Devarkar SC Budding CR Pathirage C Kavoor A Herbert C Limbach PA . Structural basis for aminoacylation of cellular modified tRNALys3 by human lysyl-tRNA synthetase. Nucleic Acids Res. (2025) 53:gkaf114. doi: 10.1093/nar/gkaf114, PMID: 40036503 PMC11878792

[3] Berger KD Puthenpeedikakkal AMK Mathews DH Fu D . Structural impact of 3-methylcytosine modification on the anticodon stem-loop of a neuronally-enriched arginine tRNA. J Mol Biol. (2025) 437:169096. doi: 10.1016/j.jmb.2025.169096, PMID: 40158946 PMC12162224

[4] Del-Pozo-Rodriguez J Tilly P Lecat R Vaca HR Mosser L Brivio E . ADAT3 variants disrupt the activity of the ADAT tRNA deaminase complex and impair neuronal migration. Brain: J Neurol. (2025) 148:3407–21. doi: 10.1093/brain/awaf109, PMID: 40120092 PMC12404733

[5] Jia H Zhang L . tRNA-derived small RNAs in disease immunity. Theranostics. (2025) 15:245–57. doi: 10.7150/thno.102650, PMID: 39744232 PMC11667222

[6] Thalalla Gamage S Khoogar R Howpay Manage S DaRos JT Crawford MC Georgeson J . Transfer RNA acetylation regulates *in vivo* mammalian stress signaling. Sci Adv. (2025) 11:eads2923. doi: 10.1126/sciadv.ads2923, PMID: 40106564 PMC11922055

[7] Kapur M Molumby MJ Guzman C Heinz S Ackerman SL . Cell-type-specific expression of tRNAs in the brain regulates cellular homeostasis. Neuron. (2024) 112:1397–415.e6. doi: 10.1016/j.neuron.2024.01.028, PMID: 38377989 PMC11065635

[8] Shulman D Dubnov S Zorbaz T Madrer N Paldor I Bennett DA . Sex-specific declines in cholinergic-targeting tRNA fragments in the nucleus accumbens in Alzheimer's disease. Alzheimer's Dementia. (2023) 19:5159–72. doi: 10.1002/alz.13095, PMID: 37158312 PMC10632545

[9] Cotter M Quinn SM Fearon U Ansboro S Rakovic T Southern JM . A new class of 7-deazaguanine agents targeting autoimmune diseases: dramatic reduction of synovial fibroblast IL-6 production from human rheumatoid arthritis patients and improved performance against murine experimental autoimmune encephalomyelitis. RSC Medicinal Chem. (2024) 15:1556–64. doi: 10.1039/d4md00028e, PMID: 38784475 PMC11110761

[10] Yuan C Liu X Cai S Zhang L Guo R Jia Z . Secreted aminoacyl-tRNA synthetase-interacting multifunctional protein-1 (AIMP1) is a promising predictor for the severity of acute AQP4-IgG positive neuromyelitis optica spectrum disorder. Multiple Sclerosis Related Disord. (2023) 70:104504. doi: 10.1016/j.msard.2023.104504, PMID: 36623394

[11] Zhang WG Zheng XR Yao Y Sun WJ Shao BZ . The role of NLRP3 inflammasome in multiple sclerosis: pathogenesis and pharmacological application. Front Immunol. (2025) 16:1572140. doi: 10.3389/fimmu.2025.1572140, PMID: 40242770 PMC11999851

[12] Jakimovski D Bittner S Zivadinov R Morrow SA Benedict RH Zipp F . Multiple sclerosis. Lancet (London England). (2024) 403:183–202. doi: 10.1016/s0140-6736(23)01473-3, PMID: 37949093

[13] Dong X Li Q Li R Li Y Jin F Li H . Inhibition of tRF- 02514 in extracellular vesicles preserves microglia pyroptosis and protects against parkinson’s disease. Mol Neurobiol. (2025) 62:11047–63. doi: 10.1007/s12035-025-04925-2, PMID: 40254704 PMC12367836

[14] Ferrari R Rivetti C Acker J Dieci G . Distinct roles of transcription factors TFIIIB and TFIIIC in RNA polymerase III transcription reinitiation. Proc Natl Acad Sci United States America. (2004) 101:13442–7. doi: 10.1073/pnas.0403851101, PMID: 15347814 PMC518776

[15] Van Breugel ME Gerber A Van Leeuwen F . The choreography of chromatin in RNA polymerase III regulation. Biochem Soc Trans. (2024) 52:1173–89. doi: 10.1042/bst20230770, PMID: 38666598 PMC11346459

[16] Schmidt CA Matera AG . tRNA introns: Presence, processing, and purpose. Wiley Interdiscip Rev RNA. (2020) 11:e1583. doi: 10.1002/wrna.1583, PMID: 31883233

[17] Popow J Englert M Weitzer S Schleiffer A Mierzwa B Mechtler K . HSPC117 is the essential subunit of a human tRNA splicing ligase complex. Sci (New York NY). (2011) 331:760–4. doi: 10.1126/science.1197847, PMID: 21311021

[18] Paushkin SV Patel M Furia BS Peltz SW Trotta CR . Identification of a human endonuclease complex reveals a link between tRNA splicing and pre-mRNA 3' end formation. Cell. (2004) 117:311–21. doi: 10.1016/s0092-8674(04)00342-3, PMID: 15109492

[19] Borsook H . A rate-governing reaction of protein synthesis. Nature. (1958) 182:1006–7. doi: 10.1038/1821006a0, PMID: 13590203

[20] Giegé R Jühling F Pütz J Stadler P Sauter C Florentz C . Structure of transfer RNAs: similarity and variability. Wiley Interdiscip Rev RNA. (2012) 3:37–61. doi: 10.1002/wrna.103, PMID: 21957054

[21] Quigley GJ Rich A . Structural domains of transfer RNA molecules. Sci (New York NY). (1976) 194:796–806. doi: 10.1126/science.790568, PMID: 790568

[22] Rich A RajBhandary UL . Transfer RNA: molecular structure, sequence, and properties. Annu Rev Biochem. (1976) 45:805–60. doi: 10.1146/annurev.bi.45.070176.004105, PMID: 60910

[23] Zhang J . Recognition of the tRNA structure: Everything everywhere but not all at once. Cell Chem Biol. (2024) 31:36–52. doi: 10.1016/j.chembiol.2023.12.008, PMID: 38159570 PMC10843564

[24] Crick F . Central dogma of molecular biology. Nature. (1970) 227:561–3. doi: 10.1038/227561a0, PMID: 4913914

[25] Ramakrishnan V . Ribosome structure and the mechanism of translation. Cell. (2002) 108:557–72. doi: 10.1016/s0092-8674(02)00619-0, PMID: 11909526

[26] Giegé R Eriani G . The tRNA identity landscape for aminoacylation and beyond. Nucleic Acids Res. (2023) 51:1528–70. doi: 10.1093/nar/gkad007, PMID: 36744444 PMC9976931

[27] Rubio Gomez MA Ibba M . Aminoacyl-tRNA synthetases. RNA (New York NY). (2020) 26:910–36. doi: 10.1261/rna.071720.119, PMID: 32303649 PMC7373986

[28] Ray PS Arif A Fox PL . Macromolecular complexes as depots for releasable regulatory proteins. Trends Biochem Sci. (2007) 32:158–64. doi: 10.1016/j.tibs.2007.02.003, PMID: 17321138

[29] Zhou Z Sun B Huang S Yu D Zhang X . Roles of aminoacyl-tRNA synthetase-interacting multi-functional proteins in physiology and cancer. Cell Death Dis. (2020) 11:579. doi: 10.1038/s41419-020-02794-2, PMID: 32709848 PMC7382500

[30] Schuntermann DB Jaskolowski M Reynolds NM Vargas-Rodriguez O . The central role of transfer RNAs in mistranslation. J Biol Chem. (2024) 300:107679. doi: 10.1016/j.jbc.2024.107679, PMID: 39154912 PMC11415595

[31] Torres AG Reina O Stephan-Otto Attolini C Ribas de Pouplana L . Differential expression of human tRNA genes drives the abundance of tRNA-derived fragments. Proc Natl Acad Sci U.S.A. (2019) 116:8451–6. doi: 10.1073/pnas.1821120116, PMID: 30962382 PMC6486751

[32] Kim HK Fuchs G Wang S Wei W Zhang Y Park H . A transfer-RNA-derived small RNA regulates ribosome biogenesis. Nature. (2017) 552:57–62. doi: 10.1038/nature25005, PMID: 29186115 PMC6066594

[33] Lai H Feng N Zhai Q . Discovery of the major 15–30 nt mammalian small RNAs, their biogenesis and function. Nat Commun. (2023) 14:5796. doi: 10.1038/s41467-023-41554-6, PMID: 37723159 PMC10507107

[34] Gong M Deng Y Xiang Y Ye D . The role and mechanism of action of tRNA-derived fragments in the diagnosis and treatment of Malignant tumors. Cell Communication Signaling: CCS. (2023) 21:62. doi: 10.1186/s12964-023-01079-3, PMID: 36964534 PMC10036988

[35] Gan M Lei Y Wang K Wang Y Liao T Ma J . A dataset of hidden small non-coding RNA in the testis of heat-stressed models revealed by Pandora-seq. Sci Data. (2024) 11:747. doi: 10.1038/s41597-024-03612-6, PMID: 38982138 PMC11233633

[36] Zhou M He X Zhang J Mei C Zhong B Ou C . tRNA-derived small RNAs in human cancers: roles, mechanisms, and clinical application. Mol Cancer. (2024) 23:76. doi: 10.1186/s12943-024-01992-2, PMID: 38622694 PMC11020452

[37] Zhu L Ge J Li T Shen Y Guo J . tRNA-derived fragments and tRNA halves: The new players in cancers. Cancer Lett. (2019) 452:31–7. doi: 10.1016/j.canlet.2019.03.012, PMID: 30905816

[38] Ivanov P Emara MM Villen J Gygi SP Anderson P . Angiogenin-induced tRNA fragments inhibit translation initiation. Mol Cell. (2011) 43:613–23. doi: 10.1016/j.molcel.2011.06.022, PMID: 21855800 PMC3160621

[39] Yamasaki S Ivanov P Hu GF Anderson P . Angiogenin cleaves tRNA and promotes stress-induced translational repression. J Cell Biol. (2009) 185:35–42. doi: 10.1083/jcb.200811106, PMID: 19332886 PMC2700517

[40] Luan N Mu Y Mu J Chen Y Ye X Zhou Q . Dicer1 promotes colon cancer cell invasion and migration through modulation of tRF-20-MEJB5Y13 expression under hypoxia. Front Genet. (2021) 12:638244. doi: 10.3389/fgene.2021.638244, PMID: 33763118 PMC7982525

[41] Lorenz C Lünse CE Mörl M . tRNA modifications: impact on structure and thermal adaptation. Biomolecules. (2017) 7:35. doi: 10.3390/biom7020035, PMID: 28375166 PMC5485724

[42] Fergus C Barnes D Alqasem MA Kelly VP . The queuine micronutrient: charting a course from microbe to man. Nutrients. (2015) 7:2897–929. doi: 10.3390/nu7042897, PMID: 25884661 PMC4425180

[43] Farkas WR Jacobson KB Katze JR . Substrate and inhibitor specificity of tRNA-guanine ribosyltransferase. Biochim Biophys Acta. (1984) 781:64–75. doi: 10.1016/0167-4781(84)90124-6, PMID: 6696916

[44] Müller M Legrand C Tuorto F Kelly VP Atlasi Y Lyko F . Queuine links translational control in eukaryotes to a micronutrient from bacteria. Nucleic Acids Res. (2019) 47:3711–27. doi: 10.1093/nar/gkz063, PMID: 30715423 PMC6468285

[45] Tuorto F Legrand C Cirzi C Federico G Liebers R Müller M . Queuosine-modified tRNAs confer nutritional control of protein translation. EMBO J. (2018) 37:e99777. doi: 10.15252/embj.201899777, PMID: 30093495 PMC6138434

[46] Rak R Polonsky M Eizenberg-Magar I Mo Y Sakaguchi Y Mizrahi O . Dynamic changes in tRNA modifications and abundance during T cell activation. Proc Natl Acad Sci United States America. (2021) 118:e2106556118. doi: 10.1073/pnas.2106556118, PMID: 34642250 PMC8594584

[47] Nakai M Hase H Zhao Y Okawa K Honda K Ikuma K . RNA-modifying enzyme Alkbh8 is involved in mouse embryonic development. iScience. (2024) 27:110777. doi: 10.1016/j.isci.2024.110777, PMID: 39280612 PMC11402254

[48] Xiong QP Li J Li H Huang ZX Dong H Wang ED . Human TRMT1 catalyzes m(2)G or m(2)(2)G formation on tRNAs in a substrate-dependent manner. Sci China Life Sci. (2023) 66:2295–309. doi: 10.1007/s11427-022-2295-0, PMID: 37204604

[49] Ontiveros RJ Shen H Stoute J Yanas A Cui Y Zhang Y . Coordination of mRNA and tRNA methylations by TRMT10A. Proc Natl Acad Sci United States America. (2020) 117:7782–91. doi: 10.1073/pnas.1913448117, PMID: 32213595 PMC7149399

[50] Chujo T Tomizawa K . Human transfer RNA modopathies: diseases caused by aberrations in transfer RNA modifications. FEBS J. (2021) 288:7096–122. doi: 10.1111/febs.15736, PMID: 33513290 PMC9255597

[51] Delaunay S Helm M Frye M . RNA modifications in physiology and disease: towards clinical applications. Nat Rev Genet. (2024) 25:104–22. doi: 10.1038/s41576-023-00645-2, PMID: 37714958

[52] Shaheen R Abdel-Salam GM Guy MP Alomar R Abdel-Hamid MS Afifi HH . Mutation in WDR4 impairs tRNA m(7)G46 methylation and causes a distinct form of microcephalic primordial dwarfism. Genome Biol. (2015) 16:210. doi: 10.1186/s13059-015-0779-x, PMID: 26416026 PMC4587777

[53] Nagayoshi Y Chujo T Hirata S Nakatsuka H Chen CW Takakura M . Loss of Ftsj1 perturbs codon-specific translation efficiency in the brain and is associated with X-linked intellectual disability. Sci Adv. (2021) 7:eabf3072. doi: 10.1126/sciadv.abf3072, PMID: 33771871 PMC7997516

[54] Freude K Hoffmann K Jensen LR Delatycki MB Des Portes V Moser B . Mutations in the FTSJ1 gene coding for a novel S-adenosylmethionine-binding protein cause nonsyndromic X-linked mental retardation. Am J Hum Genet. (2004) 75:305–9. doi: 10.1086/422507, PMID: 15162322 PMC1216064

[55] Baranowski W Dirheimer G Jakowicki JA Keith G . Deficiency of queuine, a highly modified purine base, in transfer RNAs from primary and metastatic ovarian Malignant tumors in women. Cancer Res. (1994) 54:4468–71., PMID: 8044797

[56] Goverman J . Autoimmune T cell responses in the central nervous system. Nat Rev Immunol. (2009) 9:393–407. doi: 10.1038/nri2550, PMID: 19444307 PMC2813731

[57] Varghese S Cotter M Chevot F Fergus C Cunningham C Mills KH . *In vivo* modification of tRNA with an artificial nucleobase leads to full disease remission in an animal model of multiple sclerosis. Nucleic Acids Res. (2017) 45:2029–39. doi: 10.1093/nar/gkw847, PMID: 28204548 PMC5389723

[58] Cotter M Varghese S Chevot F Fergus C Kelly VP Connon SJ . Queuine analogues incorporating the 7-aminomethyl-7-deazaguanine core: structure-activity relationships in the treatment of experimental autoimmune encephalomyelitis. ChemMedChem. (2023) 18:e202300207. doi: 10.1002/cmdc.202300207, PMID: 37350546

[59] Fergus C Al-Qasem M Cotter M McDonnell CM Sorrentino E Chevot F . The human tRNA-guanine transglycosylase displays promiscuous nucleobase preference but strict tRNA specificity. Nucleic Acids Res. (2021) 49:4877–90. doi: 10.1093/nar/gkab289, PMID: 34009357 PMC8136771

[60] Martin S Allan KC Pinkard O Sweet T Tesar PJ Coller J . Oligodendrocyte differentiation alters tRNA modifications and codon optimality-mediated mRNA decay. Nat Commun. (2022) 13:5003. doi: 10.1038/s41467-022-32766-3, PMID: 36008413 PMC9411196

[61] Vilardo E Amman F Toth U Kotter A Helm M Rossmanith W . Functional characterization of the human tRNA methyltransferases TRMT10A and TRMT10B. Nucleic Acids Res. (2020) 48:6157–69. doi: 10.1093/nar/gkaa353, PMID: 32392304 PMC7293042

[62] Tresky R Miyamoto Y Nagayoshi Y Yabuki Y Araki K Takahashi Y . TRMT10A dysfunction perturbs codon translation of initiator methionine and glutamine and impairs brain functions in mice. Nucleic Acids Res. (2024) 52:9230–46. doi: 10.1093/nar/gkae520, PMID: 38950903 PMC11347157

[63] Efthymiou S Leo CP Deng C Lin SJ Maroofian R Lin R . Biallelic pathogenic variants in TRMT1 disrupt tRNA modification and induce a neurodevelopmental disorder. Am J Hum Genet. (2025) 112:1117–38. doi: 10.1016/j.ajhg.2025.03.015, PMID: 40245862 PMC12120178

[64] McGinley MP Goldschmidt CH Rae-Grant AD . Diagnosis and treatment of multiple sclerosis: A review. JAMA. (2021) 325:765–79. doi: 10.1001/jama.2020.26858, PMID: 33620411

[65] Harding K Williams O Willis M Hrastelj J Rimmer A Joseph F . Clinical outcomes of escalation vs early intensive disease-modifying therapy in patients with multiple sclerosis. JAMA Neurol. (2019) 76:536–41. doi: 10.1001/jamaneurol.2018.4905, PMID: 30776055 PMC6515582

[66] Chiou NT Kageyama R Ansel KM . Selective Export into Extracellular Vesicles and Function of tRNA Fragments during T Cell Activation. Cell Rep. (2018) 25:3356–70.e4. doi: 10.1016/j.celrep.2018.11.073, PMID: 30566862 PMC6392044

[67] Needhamsen M Khoonsari PE Zheleznyakova GY Piket E Hagemann-Jensen M Han Y . Integration of small RNAs from plasma and cerebrospinal fluid for classification of multiple sclerosis. Front Genet. (2022) 13:1042483. doi: 10.3389/fgene.2022.1042483, PMID: 36468035 PMC9713411

[68] Malhotra S Miras MCM Pappolla A Montalban X Comabella M . Liquid biopsy in neurological diseases. Cells. (2023) 12:1911. doi: 10.3390/cells12141911, PMID: 37508574 PMC10378132

[69] Hogg MC Raoof R El Naggar H Monsefi N Delanty N O'Brien DF . Elevation in plasma tRNA fragments precede seizures in human epilepsy. J Clin Invest. (2019) 129:2946–51. doi: 10.1172/jci126346, PMID: 31039137 PMC6597227

[70] McArdle H Hogg MC Bauer S Rosenow F Prehn JHM Adamson K . Quantification of tRNA fragments by electrochemical direct detection in small volume biofluid samples. Sci Rep. (2020) 10:7516. doi: 10.1038/s41598-020-64485-4, PMID: 32371908 PMC7200677

[71] Sundrud MS Koralov SB Feuerer M Calado DP Kozhaya AE Rhule-Smith A . Halofuginone inhibits TH17 cell differentiation by activating the amino acid starvation response. Sci (New York NY). (2009) 324:1334–8. doi: 10.1126/science.1172638, PMID: 19498172 PMC2803727

[72] Keller TL Zocco D Sundrud MS Hendrick M Edenius M Yum J . Halofuginone and other febrifugine derivatives inhibit prolyl-tRNA synthetase. Nat Chem Biol. (2012) 8:311–7. doi: 10.1038/nchembio.790, PMID: 22327401 PMC3281520

[73] Mazziotti V Crescenzo F Turano E Guandalini M Bertolazzo M Ziccardi S . The contribution of tumor necrosis factor to multiple sclerosis: a possible role in progression independent of relapse? J Neuroinflamm. (2024) 21:209. doi: 10.1186/s12974-024-03193-6, PMID: 39169320 PMC11340196

[74] Chai Y Lu Y Yang L Qiu J Qin C Zhang J . Identification and potential functions of tRNA-derived small RNAs (tsRNAs) in irritable bowel syndrome with diarrhea. Pharmacol Res. (2021) 173:105881. doi: 10.1016/j.phrs.2021.105881, PMID: 34509631

[75] Shen L Liao T Chen Q Lei Y Wang L Gu H . tRNA-derived small RNA, 5'tiRNA-Gly-CCC, promotes skeletal muscle regeneration through the inflammatory response. J Cachexia Sarcopenia Muscle. (2023) 14:1033–45. doi: 10.1002/jcsm.13187, PMID: 36755335 PMC10067481

[76] Abeywansha T Huang W Ye X Nawrocki A Lan X Jankowsky E . The structural basis of tRNA recognition by arginyl-tRNA-protein transferase. Nat Commun. (2023) 14:2232. doi: 10.1038/s41467-023-38004-8, PMID: 37076488 PMC10115844

[77] Zuchero JB Fu MM Sloan SA Ibrahim A Olson A Zaremba A . CNS myelin wrapping is driven by actin disassembly. Dev Cell. (2015) 34:152–67. doi: 10.1016/j.devcel.2015.06.011, PMID: 26166300 PMC4519368

[78] Palandri A Bonnet LV Farias MG Hallak ME Galiano MR . Ablation of arginyl-tRNA-protein transferase in oligodendrocytes impairs central nervous system myelination. Glia. (2022) 70:303–20. doi: 10.1002/glia.24107, PMID: 34669233

[79] Brown TL Macklin WB . The actin cytoskeleton in myelinating cells. Neurochemical Res. (2020) 45:684–93. doi: 10.1007/s11064-019-02753-0, PMID: 30847860 PMC6732044

[80] Murdock BJ Goutman SA Boss J Kim S Feldman EL . Amyotrophic lateral sclerosis survival associates with neutrophils in a sex-specific manner. Neurology(R) Neuroimmunology Neuroinflamm. (2021) 8:e953. doi: 10.1212/nxi.0000000000000953, PMID: 33531377 PMC8057067

[81] Murdock BJ Zhao B Webber-Davis IF Teener SJ Pawlowski KD Famie JP . Early immune system changes in amyotrophic lateral sclerosis correlate with later disease progression. Med (New York NY). (2025) 6:100673. doi: 10.1016/j.medj.2025.100673, PMID: 40286795 PMC12335928

[82] Kook MG Byun MR Lee SM Lee MH Lee DH Lee HB . Anti-apoptotic splicing variant of AIMP2 recover mutant SOD1-induced neuronal cell death. Mol Neurobiol. (2023) 60:145–59. doi: 10.1007/s12035-022-03073-1, PMID: 36242734

[83] Long JM Holtzman DM . Alzheimer disease: an update on pathobiology and treatment strategies. Cell. (2019) 179:312–39. doi: 10.1016/j.cell.2019.09.001, PMID: 31564456 PMC6778042

[84] van Olst L Gate D . Microglia drive amyloid-β clearance in immunized patients with Alzheimer's disease. Nat Med. (2025) 31:1418–9. doi: 10.1038/s41591-025-03677-9, PMID: 40263632

[85] Avalos J Corrochano LM Brenner S . Cysteinyl-tRNA synthetase is a direct descendant of the first aminoacyl-tRNA synthetase. FEBS Lett. (1991) 286:176–80. doi: 10.1016/0014-5793(91)80968-9, PMID: 1864365

[86] Luodan A Qu L He J Ge L Gao H Huang X . Exosomes derived from IFNγ-stimulated mesenchymal stem cells protect photoreceptors in RCS rats by restoring immune homeostasis through tsRNAs. Cell Communication Signaling: CCS. (2024) 22:543. doi: 10.1186/s12964-024-01920-3, PMID: 39538308 PMC11562488

[87] Kielian T Mayes P Kielian M . Characterization of microglial responses to Staphylococcus aureus: effects on cytokine, costimulatory molecule, and Toll-like receptor expression. J Neuroimmunology. (2002) 130:86–99. doi: 10.1016/s0165-5728(02)00216-3, PMID: 12225891

[88] Qi XH Chen P Wang YJ Zhou ZP Liu XC Fang H . Increased cysteinyl-tRNA synthetase drives neuroinflammation in Alzheimer's disease. Trans Neurodegeneration. (2024) 13:3. doi: 10.1186/s40035-023-00394-6, PMID: 38191451 PMC10773087

[89] Campoy-Campos G Solana-Balaguer J Guisado-Corcoll A Chicote-González A Garcia-Segura P Pérez-Sisqués L . RTP801 interacts with the tRNA ligase complex and dysregulates its RNA ligase activity in Alzheimer's disease. Nucleic Acids Res. (2024) 52:11158–76. doi: 10.1093/nar/gkae776, PMID: 39268577 PMC11472047

[90] Deng H Mi MT . Resveratrol attenuates Aβ25–35 caused neurotoxicity by inducing autophagy through the tyrRS-PARP1-SIRT1 signaling pathway. Neurochemical Res. (2016) 41:2367–79. doi: 10.1007/s11064-016-1950-9, PMID: 27180189

[91] Ouchi Y Yagi S Yokokura M Sakamoto M . Neuroinflammation in the living brain of Parkinson's disease. Parkinsonism Related Disord. (2009) 15:S200–4. doi: 10.1016/s1353-8020(09)70814-4, PMID: 20082990

[92] Tansey MG Wallings RL Houser MC Herrick MK Keating CE Joers V . Inflammation and immune dysfunction in Parkinson disease. Nat Rev Immunol. (2022) 22:657–73. doi: 10.1038/s41577-022-00684-6, PMID: 35246670 PMC8895080

[93] Rajan S Tryphena KP Khan S Vora L Srivastava S Singh SB . Understanding the involvement of innate immunity and the Nrf2-NLRP3 axis on mitochondrial health in Parkinson's disease. Ageing Res Rev. (2023) 87:101915. doi: 10.1016/j.arr.2023.101915, PMID: 36963313

[94] Madrer N Vaknine-Treidel S Zorbaz T Tzur Y Bennett ER Drori P . Pre-symptomatic Parkinson's disease blood test quantifying repetitive sequence motifs in transfer RNA fragments. Nat Aging. (2025) 5:868–82. doi: 10.1038/s43587-025-00851-z, PMID: 40216989 PMC12092246

[95] Winek K Lobentanzer S Nadorp B Dubnov S Dames C Jagdmann S . Transfer RNA fragments replace microRNA regulators of the cholinergic poststroke immune blockade. Proc Natl Acad Sci United States America. (2020) 117:32606–16. doi: 10.1073/pnas.2013542117, PMID: 33288717 PMC7768686

[96] Meisel C Schwab JM Prass K Meisel A Dirnagl U . Central nervous system injury-induced immune deficiency syndrome. Nat Rev Neurosci. (2005) 6:775–86. doi: 10.1038/nrn1765, PMID: 16163382

[97] Basavaraju S Mishra S Jindal R Kesavardhana S . Emerging role of ZBP1 in Z-RNA sensing, influenza virus-induced cell death, and pulmonary inflammation. mBio. (2022) 13:e0040122. doi: 10.1128/mbio.00401-22, PMID: 35587190 PMC9239214

[98] Zhang Y Ma Y Ji YK Jiang YF Li D Mu W . Co-targeting of glial activation and inflammation by tsRNA-Gln-i-0095 for treating retinal ischemic pathologies. Cell Communication Signaling: CCS. (2025) 23:18. doi: 10.1186/s12964-024-02013-x, PMID: 39794828 PMC11721595

[99] Cai H Zhang Y Meng F Li Y . Effects of spinal cord injury associated exosomes delivered tRF-41 on the progression of spinal cord injury progression. Genomics. (2024) 116:110885. doi: 10.1016/j.ygeno.2024.110885, PMID: 38866256

[100] Yang P Mustafi D Pepple KL . Immunology of retinitis pigmentosa and gene therapy-associated uveitis. Cold Spring Harbor Perspect Med. (2024) 14:a041305. doi: 10.1101/cshperspect.a041305, PMID: 37037600 PMC10562523

[101] Koureas M Nasika A Lianos AG Vontas A Kyritsi MA Voulgaridi I . Seroprevalence of west nile virus, Greece, 2020. Euro Surveillance: Bull Europeen Sur Les Maladies Transmissibles = Eur Communicable Dis Bull. (2025) 30:2400487. doi: 10.2807/1560-7917.es.2025.30.15.2400487, PMID: 40248889 PMC12007402

[102] Duerlund LS Nielsen H Bodilsen J . Current epidemiology of infectious encephalitis: a narrative review. Clin Microbiol Infection. (2025) 31:515–21. doi: 10.1016/j.cmi.2024.12.025, PMID: 39725074

[103] Clarke P Leser JS Bowen RA Tyler KL . Virus-induced transcriptional changes in the brain include the differential expression of genes associated with interferon, apoptosis, interleukin 17 receptor A, and glutamate signaling as well as flavivirus-specific upregulation of tRNA synthetases. mBio. (2014) 5:e00902–14. doi: 10.1128/mBio.00902-14, PMID: 24618253 PMC3952157

[104] Monash A Madrer N Treidel SV Israeli O Hinden L Greenberg DS . 5′LysTTT tRNA fragments support survival of botulinum-intoxicated neurons by blocking ferroptosis. Genomic Psychiatry. (2025) 1:1–17. doi: 10.61373/gp025a.0047

[105] Chen ZY Mortha A . Mitochondria in monocyte-derived cells promote tissue damage in multiple sclerosis. Nat Rev Immunol. (2025) 25:3. doi: 10.1038/s41577-024-01116-3, PMID: 39592784

[106] Liu Z Bai Y Xu B Wen H Chen K Lin J . TDP43 augments astrocyte inflammatory activity through mtDNA-cGAS-STING axis in NMOSD. J Neuroinflamm. (2025) 22:14. doi: 10.1186/s12974-025-03348-z, PMID: 39844196 PMC11756062

[107] Li B Liu F Chen X Chen T Zhang J Liu Y . FARS2 deficiency causes cardiomyopathy by disrupting mitochondrial homeostasis and the mitochondrial quality control system. Circulation. (2024) 149:1268–84. doi: 10.1161/circulationaha.123.064489, PMID: 38362779 PMC11017836

[108] Suzuki T Nagao A Suzuki T . Human mitochondrial tRNAs: biogenesis, function, structural aspects, and diseases. Annu Rev Genet. (2011) 45:299–329. doi: 10.1146/annurev-genet-110410-132531, PMID: 21910628

[109] Suzuki T Yashiro Y Kikuchi I Ishigami Y Saito H Matsuzawa I . Complete chemical structures of human mitochondrial tRNAs. Nat Commun. (2020) 11:4269. doi: 10.1038/s41467-020-18068-6, PMID: 32859890 PMC7455718

[110] Loher P Telonis AG Rigoutsos I . MINTmap: fast and exhaustive profiling of nuclear and mitochondrial tRNA fragments from short RNA-seq data. Sci Rep. (2017) 7:41184. doi: 10.1038/srep41184, PMID: 28220888 PMC5318995

[111] Telonis AG Loher P Magee R Pliatsika V Londin E Kirino Y . tRNA fragments show intertwining with mRNAs of specific repeat content and have links to disparities. Cancer Res. (2019) 79:3034–49. doi: 10.1158/0008-5472.can-19-0789, PMID: 30996049 PMC6571059

[112] Andalib S Talebi M Sakhinia E Farhoudi M Sadeghi-Bazargani H Gjedde A . Lack of association between mitochondrial DNA G15257A and G15812A variations and multiple sclerosis. J Neurological Sci. (2015) 356:102–6. doi: 10.1016/j.jns.2015.06.022, PMID: 26233806

[113] Mayr-Wohlfart U Paulus C Henneberg A Rödel G . Mitochondrial DNA mutations in multiple sclerosis patients with severe optic involvement. Acta Neurologica Scandinavica. (1996) 94:167–71. doi: 10.1111/j.1600-0404.1996.tb07048.x, PMID: 8899049

[114] Kalman B Lublin FD Alder H . Mitochondrial DNA mutations in multiple sclerosis. Multiple Sclerosis (Houndmills Basingstoke England). (1995) 1:32–6. doi: 10.1177/135245859500100106, PMID: 9345467

[115] Pena JA Lotze T Yang Y Umana L Walkiewicz M Hunter JV . Methionyl-tRNA formyltransferase (MTFMT) deficiency mimicking acquired demyelinating disease. J Child Neurol. (2016) 31:215–9. doi: 10.1177/0883073815587946, PMID: 26060307

[116] Scheper GC Van Der Klok T Van Andel RJ Van Berkel CG Sissler M Smet J . Mitochondrial aspartyl-tRNA synthetase deficiency causes leukoencephalopathy with brain stem and spinal cord involvement and lactate elevation. Nat Genet. (2007) 39:534–9. doi: 10.1038/ng2013, PMID: 17384640

[117] Isohanni P Linnankivi T Buzkova J Lönnqvist T Pihko H Valanne L . DARS2 mutations in mitochondrial leucoencephalopathy and multiple sclerosis. J Med Genet. (2010) 47:66–70. doi: 10.1136/jmg.2009.068221, PMID: 19592391

[118] Wolf NI Toro C Kister I Latif KA Leventer R Pizzino A . DARS-associated leukoencephalopathy can mimic a steroid-responsive neuroinflammatory disorder. Neurology. (2015) 84:226–30. doi: 10.1212/wnl.0000000000001157, PMID: 25527264 PMC4335995

[119] Taylor RW Chinnery PF Bates MJ Jackson MJ Johnson MA Andrews RM . A novel mitochondrial DNA point mutation in the tRNA(Ile) gene: studies in a patient presenting with chronic progressive external ophthalmoplegia and multiple sclerosis. Biochem Biophys Res Commun. (1998) 243:47–51. doi: 10.1006/bbrc.1997.8055, PMID: 9473477

[120] Johnson BS Farkas D El-Mergawy R Adair JA Elhance A Eltobgy M . Targeted degradation of extracellular mitochondrial aspartyl-tRNA synthetase modulates immune responses. Nat Commun. (2024) 15:6172. doi: 10.1038/s41467-024-50031-7, PMID: 39039092 PMC11263397

[121] Vaknine Treidel S Lobmaier SM Sharma R Madrer N Dubnov S Shulman D . Maternal prenatal stress induces sex-dependent changes in tRNA fragment families and cholinergic pathways in newborns. Mol Psychiatry. (2025) 30:4307–19. doi: 10.1038/s41380-025-03011-2, PMID: 40188313 PMC12339370

[122] Pedder JH Sonabend AM Cearns MD Michael BD Zakaria R Heimberger AB . Crossing the blood-brain barrier: emerging therapeutic strategies for neurological disease. Lancet Neurol. (2025) 24:246–60. doi: 10.1016/s1474-4422(24)00476-9, PMID: 39862873 PMC12242972

[123] Qiao R Jia Q Hüwel S Xia R Liu T Gao F . Receptor-mediated delivery of magnetic nanoparticles across the blood-brain barrier. ACS Nano. (2012) 6:3304–10. doi: 10.1021/nn300240p, PMID: 22443607

